# Supplementary material for: Behavioral innovation promotes alien bird invasions
Source: Innovation (Camb). 2021 Sep 11;2(4):100167. doi: 10.1016/j.xinn.2021.100167 (PMC8488305; doi:10.1016/j.xinn.2021.100167)
Supplement: Document S1. Tables S1–S3 [file mmc1.pdf]

The Innovation, Volume *2*

Supplemental Information

Behavioral innovation promotes alien bird invasions

Daiping Wang and Xuan Liu

Supplemental Information

Table S1. Linear mixed-effects model testing associations between establishment success rate and innovation along with other variables based on 247 species. Here, note that 'innovation rate' (i.e., the sum of 'consumer innovations' and 'technical innovations') is added as a fixed effect.

	Estimate		
	(β ± SE)	t	p
Random effects:			
Phylogeny (n =247)	0.61 (0.34-0.76)		
Fixed effects:			
Intercept	0.566 ± 0.146	3.9	——
Innovation rate	0.070 ± 0.023	3.1	0.002
Established populations	-0.019 ± 0.024	-0.8	0.435
Reproductive investment	-0.018 ± 0.033	-0.5	0.587
Propagule pressure	0.074 ± 0.021	3.6	<0.001
Different to native	-0.055 ± 0.026	-2.1	0.033
Habitat generalism	0.033 ± 0.023	1.4	0.154

Table S2. Linear mixed-effects model testing associations between establishment success rate and innovation along with other variables based on 247 species. Here, note that ‘Consumer innovations’ (i.e., the number of novel feeding behaviors) is added as a fixed effect.

	Estimate (β ± SE)	t	p
Random effects:			
Phylogeny (n =247)	0.61 (0.34-0.76)		
Fixed effects:			
Intercept	0.567 ± 0.147	3.9	——
Consumer innovations	0.053 ± 0.021	2.5	0.012
Established populations	-0.022 ± 0.024	-0.9	0.369
Reproductive investment	-0.018 ± 0.034	-0.5	0.601
Propagule pressure	0.073 ± 0.021	3.5	<0.001
Different to native	-0.058 ± 0.026	-2.3	0.025
Habitat generalism	0.030 ± 0.023	1.3	0.197

Table S3. Linear mixed-effects model testing associations between establishment success rate and innovation along with other variables based on 247 species. Here, note that ‘Technical innovations’ (i.e., the number of novel searching and handling technique behaviors) is added as a fixed effect.

	Estimate ($\beta \pm SE$)	t	p
Random effects:			
Phylogeny (n =247)	0.61 (0.33-0.76)		
Fixed effects:			
Intercept	0.565 ± 0.145	3.9	——
Technical innovations	0.076 ± 0.024	3.2	0.0018
Established populations	-0.016 ± 0.024	-0.7	0.515
Reproductive investment	-0.021 ± 0.033	-0.6	0.526
Propagule pressure	0.077 ± 0.020	3.7	<0.001
Different to native	-0.053 ± 0.026	-2.1	0.04
Habitat generalism	0.037 ± 0.023	1.6	0.102
