# Supplementary material for: Behavioral innovation promotes alien bird invasions
Source: Innovation (Camb). 2021 Sep 11;2(4):100167. doi: 10.1016/j.xinn.2021.100167 (PMC8488305; doi:10.1016/j.xinn.2021.100167)
Supplement: Document S2. Article plus supplemental information [file mmc2.pdf]

Report

Behavioral innovation promotes alien bird invasions

Daiping Wang[1] and Xuan Liu[1,2,*]
*Correspondence: liuxuan@ioz.ac.cn
Received: January 22, 2021; Accepted: September 10, 2021; Published Online: September 11, 2021; https://doi.org/10.1016/j.xinn.2021.100167
© 2021 The Author(s). This is an open access article under the CC BY license (http://creativecommons.org/licenses/by/4.0/).

Graphical abstract

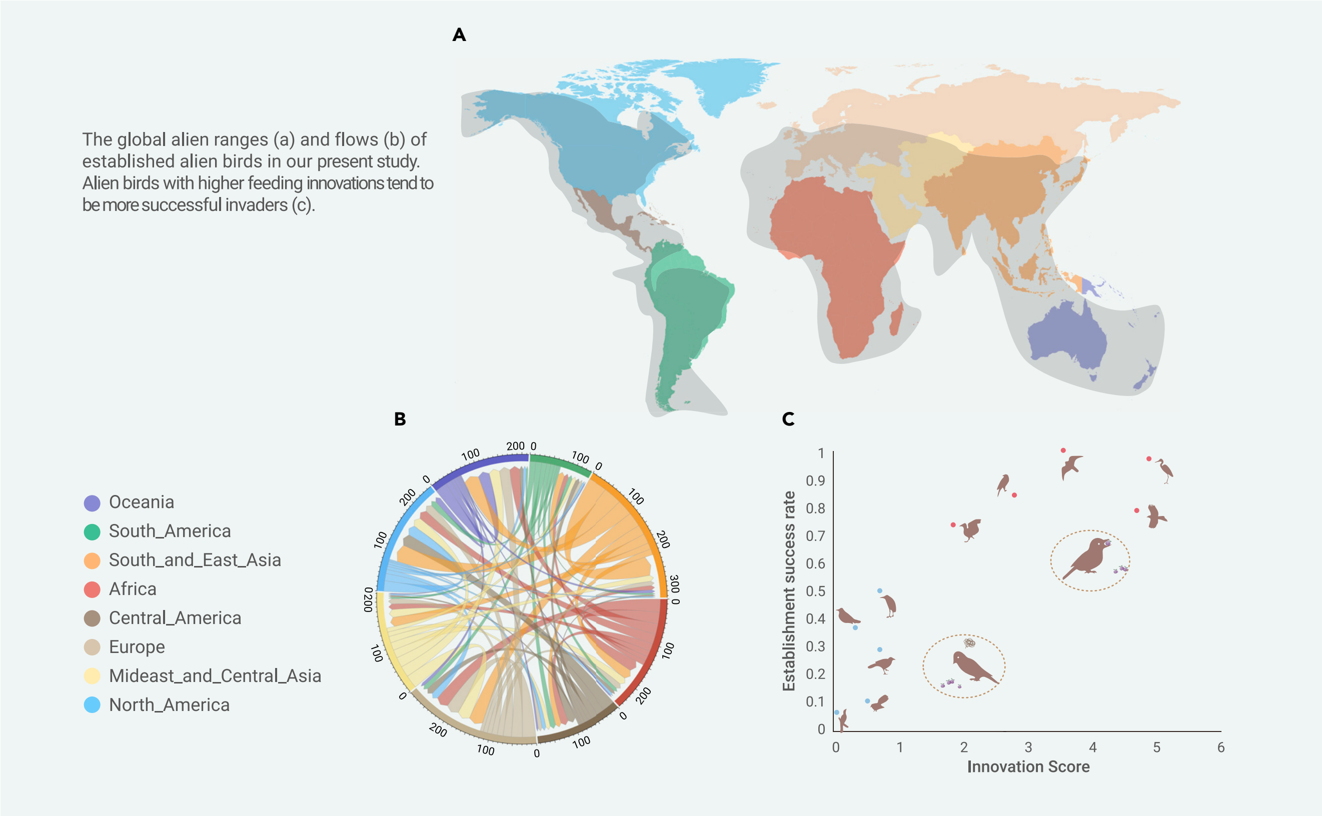

Public summary

- The role of behavioral innovation in alien bird establishment is analyzed worldwide
- Behavioral innovation is a key factor facilitating alien bird establishment success
- Technical innovation is more influential than consumer innovation in this process

Behavioral innovation promotes alien bird invasions

Daiping Wang[1] **and Xuan Liu**[1,2,*]

[1]Key Laboratory of Animal Ecology and Conservation Biology, Institute of Zoology, Chinese Academy of Sciences, 1 Beichen West Road, Chaoyang, Beijing 100101, China

[2]University of Chinese Academy of Sciences, Beijing 100049, China

*Correspondence: liuxuan@ioz.ac.cn

Received: January 22, 2021; Accepted: September 10, 2021; Published Online: September 11, 2021; https://doi.org/10.1016/j.xinn.2021.100167

© 2021 The Author(s). This is an open access article under the CC BY license (http://creativecommons.org/licenses/by/4.0/).

Citation: Wang D. and Liu X. (2021). Behavioral innovation promotes alien bird invasions. The Innovation **2(4)**, 100167.

Behavioral innovation is believed to represent the ability of species to adapt to novel environments and to thus affect the observed establishment success of alien species in a new range. However, the relative importance of behavioral innovation in explaining alien species establishment among key event-, location-, and species-level factors remains poorly evaluated. In addition, the effects of technical innovation in food searching and handling techniques and consumer innovation in the use of new foods on establishment success are not clear. Here, based on a global dataset including information on 247 species across 9,899 successful and 2,370 failed introduction events spanning 199 countries or regions worldwide, we show that the behavioral innovation rate is a key factor facilitating alien bird establishment success after considering propagule pressure, climate matching, historical invasional meltdown, and life-history traits. Furthermore, we find that technical innovation is more influential than consumer innovation in explaining establishment success. Our results contribute to a deeper understanding of the effect of behavioral innovation on the establishment success of alien species in new ranges and may help predict the response of both native and alien species to accelerating global change during the Anthropocene.

Keywords: biological invasion; behavioral innovation; global change; behavioral plasticity; alien bird

INTRODUCTION

With the increasing introduction of alien species in the era of globalization,[1] biological invasion has been regarded as a major global change component causing rapid biodiversity decline, economic damage, and public health concerns.[2] Understanding why some alien species are more successful than others when introduced into the same regions is crucial for predicting new invasion events and developing effective prevention strategies at early stages.[3] Among the different invasion determinants, species traits have always been a central factor.[4] This is particularly true for behavioral innovation, which reflects the ability of species to cope with novel environments in their invaded ranges, and there have been great exploratory efforts addressing the effect of such innovation on the observed variation in alien species establishment probability.[5–8] However, the generality and relative importance of behavioral innovation in the establishment success of alien species remain difficult to identify for several reasons. First, most previous studies were based on indirect behavioral innovation surrogates such as brain size.[7,8] Nevertheless, brain size may be related to many components of species traits, and it has been shown that brain size mainly works through behavioral plasticity variables; thus, behavioral innovation is a more direct variable reflecting the adaptation ability of species in response to changing environments.[6] Second, for studies based on behavioral innovation variables, previous evidence was largely obtained at the continental or country scale, such as data on alien birds in New Zealand.[5] Third, global studies using behavioral innovation as the variable[6] have been based on relatively limited sample sizes compared with those on currently known established alien birds.[9] In addition, these previous studies did not consider some important climatic and biotic predictors, notably environmental similarity with that of the species' native distributions, reflecting the climatic suitability hypothesis,[10] and the presence of other alien groups, reflecting the invasional meltdown hypothesis,[11] both of which have been identified as primary hypotheses of alien species establishment.[12] Therefore, the documented correlations between these key variables and establishment success may confound the behavioral innovation effect, and we thus know little about the relative importance of behavioral innovation when incorporating these key predictor variables together. Furthermore, there are different innovation types, including consumer innovation, such as the use of new foods in a species' diet, and technical innovation, such as food searching and handling techniques.[13] Whether there are differences in the effects of consumer and technical innovations on establishment success remains largely unknown. Finally, certain behavioral features of species may be highly phylogenetically conserved;[14] therefore, more elaborate phylogenetic methods are needed to control potential phylogenetic pseudoreplication of ecological characteristics among species.[15]

Alien bird invasions provide an ideal model system and unique opportunity to address these scientific questions worldwide. First, relatively accurate recordings of global avian establishment status comprising 27,723 population records spanning 971 alien bird species across more than 200 countries and territories that are updated in a timely manner are available.[9] Crucially, these data provide quantitative information on important factors affecting population establishment, such as propagule pressure; environmental differences between the native and alien ranges; the richness of historically established alien species across multiple taxonomic groups prior to the arrival of the bird invaders; species traits representing population growth, such as reproductive investment; and habitat generalism.[12] Furthermore, a comprehensive and global dataset quantifying the behavioral innovation of 8,645 birds was recently developed,[13] making it possible to test the generality of the behavioral plasticity hypothesis using more alien birds than ever before at the global scale. Behavioral innovation is regarded as an ideal metric for measuring behavioral plasticity ability when individuals encounter new abiotic and biotic selection pressures in novel environments.[13] Here, based on data for 247 alien bird invaders (12,269 population records) with available behavioral innovation information in 199 countries or regions across seven global biogeographic regions and five other crucial determinants of establishment success of alien birds,[12] we aim to assess the relative importance of behavioral innovation, propagule pressure, climate similarity, invasional meltdown, reproductive investment, and habitat generalism on alien bird establishment success across the globe. We further distinguish the relative roles of technical innovation and consumer innovation in predicting establishment success. We hypothesize that behavioral innovation may be an important variable in predicting the probability of alien bird establishment success after accounting for all previously pivotal confounding variables and phylogenetic autocorrelation, and there might be variations in the relative contributions of technical innovation and consumer innovation to establishment success.

RESULTS

Among the 247 species, we found that a couple of taxonomic groups tended to be more successful regarding their establishment in a new range (Figure 1). Typically, groups with a high success rate of establishment were (1) some goose and swan species, such as the pink-footed goose (*Anser brachyrhynchus*), red-breasted goose (*Branta ruficollis*), and trumpeter

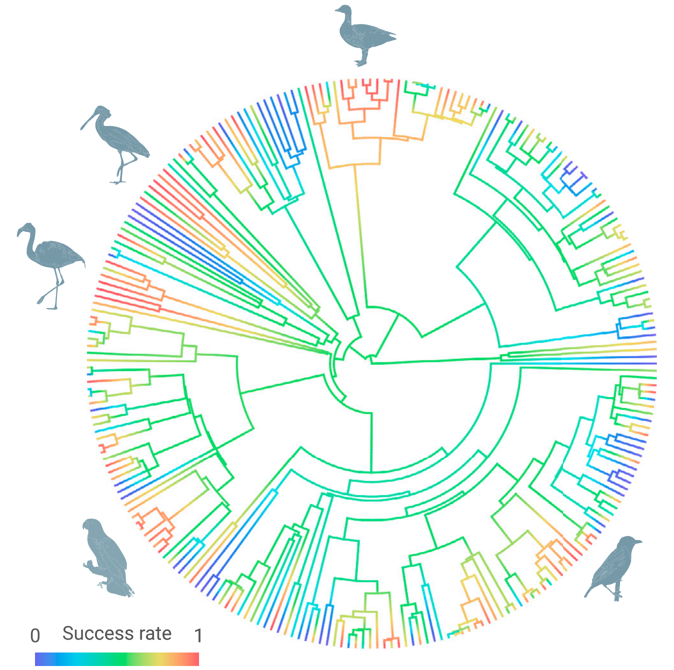

Figure 1. Phylogenetic distribution of the establishment success rate (from 0 to 1: blue to red) for 247 alien bird species Silhouettes (retrieved from www.phylopic.org) represent some typical taxonomic groups with a high establishment success rate in a new range.

swan (*Cygnus buccinator*); (2) the African sacred ibis (*Threskiornis aethiopicus*) and African spoonbill (*Platalea alba*); (3) some flamingo species, such as the greater flamingo (*Phoenicopterus roseus*) and lesser flamingo (*Phoeniconaias minor*); (4) some Amazon species, such as the yellow-headed Amazon (*Amazona oratrix*) and red-crowned Amazon (*Amazona viridigenalis*); and (5) some weaver species, such as the village weaver (*Ploceus cucullatus*) and streaked weaver (*Ploceus manyar*) (Figure 1).

Linear mixed-effects models with multiple regression accounting for phylogenetic autocorrelation as a random effect (λ = 0.61; 95% confidence interval, 0.34–0.76; Table S1) showed that species innovation significantly promoted the establishment success of alien birds (t = 3.1, p = 0.002, n = 247 species; Figures 2 and 3; Table S1). Furthermore, propagule pressure (i.e., founding population size, t = 3.6, $p < 0.001$; Figure 3; Table S1) was the most important variable, and the effect size of the innovation rate was larger than that of most of the other variables, such as established populations, reproductive investment, and habitat generalism, which greatly affected the establishment success of alien birds (Figure 3; Table S1). Thus, behavioral innovation tended to be a more important factor affecting the establishment success of alien species in a new environment. More specifically, we found that both consumer and technical innovations facilitated the species' establishment success rate (consumer innovations: t = 2.5, p = 0.01. Technical innovations: t = 3.2, p = 0.0018), although technical innovations seemed to be a more influential factor (Figure 3; Tables S2 and S3). Additionally, we found a significant effect size of difference from native environment (i.e., the difference in environmental conditions between an invaded location and the native range of a species; t = −2.1, p = 0.03; Figure 3; Table S1), which was consistent with the results of a previous study.[12]

DISCUSSION

Our study based on recent alien bird invasion and foraging innovation data reveals that behavioral innovation is indeed an important factor determining alien bird establishment, indicating the generality of this phenomenon in avian invasions. However, we covered many more species and regions of the world than past studies and incorporated more key processes determining alien bird establishment. We also found that bird technical innovation in searching and handling foods is more crucial than adopting new food types in predicting establishment success. This finding is of great importance because we accounted for phylogenetic relatedness in greater detail and quantified the relative importance of each of the predictor variables, showing that behavioral innovation was the second most important variable contributing to the probability of alien bird establishment after propagule pressure, particularly in terms of the ability to develop new foraging techniques.

Behavioral innovation has long attracted great attention as an important attribute of successful invaders.[5,6] Our present study provides further quantitative evidence of its important effect after accounting for other key determinants at the invasion event, location, and species levels. Behavioral innovation can reflect learning, cognition, and rapid adjustment to new environments and thus plays a critical role when species invade new habitats. Specifically, the foraging innovation considered here can indicate variation in the discovery of hard-to-reach foods by alien species and changes in food availability in new ranges.[16] The critical effect of propagule pressure is not surprising because the magnitude of introduction efforts has long been regarded as a fundamental driver of alien species establishment.[17,18] Our results support the propagule pressure hypothesis, which posits that the probability of alien species establishment increases with the number of introduction events and the number of individuals involved in each introduction event[19] because high propagule pressure can overcome inbreeding depression and genetic drift due to demographic and environmental stochasticity or Allee effects.[18] We also provide results consistent with the finding of an important role of climatic similarity between native and invaded ranges in explaining alien bird establishment. These results support the climatic similarity hypothesis, which states that alien species may be more likely to establish naturalized populations in alien environments with high climatic suitability in terms of similarity with the species' native ranges, as it has been suggested that such species may be preadapted to nonnative conditions.[20] Indeed, as a fundamental ecological principle related to species distributions, climatic matching between native and alien ranges is widely recognized as a key predictor of establishment success by nonnative populations,[21] and emerging evidence across taxa has demonstrated the high conservatism of climatic niches during the invasion process.[22]

We did not detect a significant contribution of reproductive investment (brood size used here), historical alien species richness, or habitat generalism to establishment of the 247 alien birds. Reproductive investment is thought to represent the population growth capacity,[23] and this variable likely added little variation that may have been captured by other more important predictors influencing population size, such as propagule pressure. We also revealed no overall significant effect of historical alien species richness. This does not mean that an effect of interactions among congeneric invaders is absent but may demonstrate the complex interactions among cooccurring invasive animals,[24,25] which is dependent on a number of factors, such as relative population density, functional equivalence, and trophic position.[26] For instance, although the invasional meltdown hypothesis suggests that there tends to be a positive relationship between the probability of establishment of one alien species and the number of prior successful invaders that can help reduce predators or competitors,[27] the naturalization hypothesis posits that the presence of sympatric species reduces the probability of establishment success owing to competition with congeners for limited resources.[28] We found no significant effect of habitat generalism on alien bird establishment, suggesting that this variable might be correlated with other more important variables, such as behavioral innovation, which represents the ability to adapt in response to various abiotic and biotic selection pressures.[29] Indeed, we found that using new foods (i.e., consumer innovation) was a significant variable affecting alien bird establishment, supporting the idea that opportunistic omnivores that can use different types of foods, especially new foods in nonnative ranges, are particularly likely to be successful invaders.[30] However, we further found that technical innovation has a larger effect size than consumer innovation, indicating that how various types of foods are used may be more crucial than the exact food per se for alien birds in terms of their ability to colonize new environments. One potential

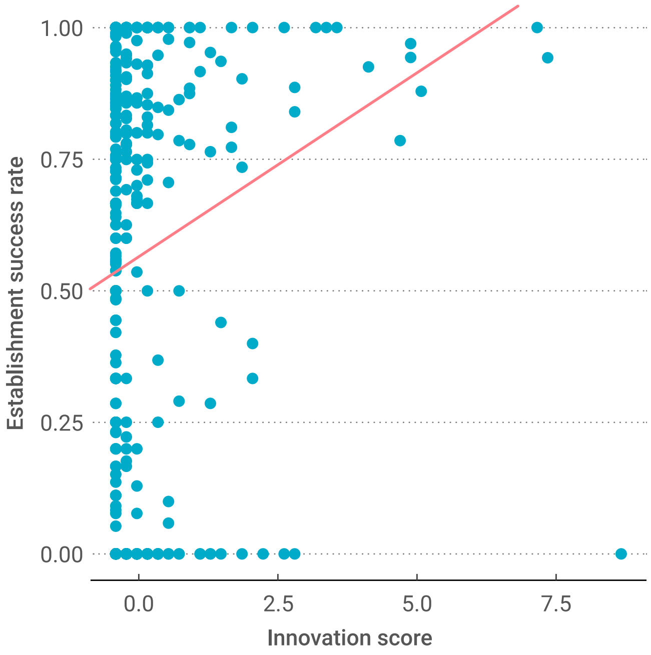

Figure 2. Establishment success rate as a function of innovation score (innovation rate) Here, the regression function (equation) is from the mixed-effects model (Table S1) with an intercept of 0.57, and the slope of the innovation score is 0.07.

explanation is that the capacity to acquire hard-to-reach foods may be key to the survival and establishment of alien species when they arrive in novel environments.

Environmental disturbance has been recognized as an important facilitator of biological invasions, as alien species are often observed taking advantage of human-modified environments.[31,32] One potential explanation for this effect is that disturbed habitats can promote nonnative species establishment by decreasing the abundance of native species and thus creating vacant niches that can be filled by invaders.[33] We argue that the important behavioral effect on alien species establishment identified here and in previous studies indicates that the habitat disturbance hypothesis might be alternatively explained by the animal behavior phenomenon in which successful invaders may have higher behavioral innovation ability when faced with disturbed habitats, which warrants additional research. We acknowledge that current evidence of the behavioral innovation hypothesis mainly stems from alien bird invasions, and its generality needs to be further tested across more taxonomic groups. In addition, existing correlative analyses based on observations must be integrated with experimental approaches to explore more causal relationships between behavioral plasticity and establishment success. For example, the observed changes in foraging behavior may be regulated by different expressions of the same physiological and genetic features in different environments.[34] Indeed, the foraging innovations that we used in the present study tended to be repeatable (intraclass coefficient = 0.36) across different regions.[13] This indicated the potential heritability of these innovation behaviors according to the theoretical prediction of quantitative genetics, which is beyond the scope of the present study but warrants further investigations when related physiological and genetic data modulating bird predatory behaviors are available. Furthermore, as alien species usually show high phenotypic plasticity and even rapid evolutionary abilities during biological invasions[35] and animal behavior has been demonstrated to be shaped by novel selection pressures,[36] further investigations of whether alien species could enhance their behavioral flexibility in varied environments in new ranges are needed. The crucial role of the behavioral innovation of alien species during establishment could not only facilitate the understanding of the potential interactions of biological invasion with other global change processes, such as land-use modifications, but also help predict the survival probability of native species in response to changing environments. Indeed, recent work showed that behavioral innovation is a good predictor of native species endangerment.[13] In response to the accelerating rate of human-induced environmental changes during the Anthropocene, comparisons of these two fields may provide valuable insights into key characteristics that allow both native and alien species to rapidly adapt to changing environments.[37]

MATERIALS AND METHODS

Establishment success rate

The establishment success data (e.g., establishment status and population location) for alien birds used in the present study are from Dyer et al.,[9] which include a database of alien bird distributions at a global scale. This database (Global Avian Invasions Atlas) comprises 27,723 population records for 971 species introduced to 230 countries and regions across all biogeographical realms. More details regarding the whole database are presented in Dyer et al.[9] In short, the raw data in this database include taxonomic, spatial, and temporal information as well as details relating to introduction events and their ultimate consequence (i.e., failure or success). For the present study, we calculated the species establishment success rate, which is the proportion of successfully established population records for each species (the number of successful invasion events divided by the total number of introduction events). By doing so, we obtained data for 688 species (18,544 population records) of this variable for further analysis.

Behavioral innovation variables

Rather than relative brain size, our present study used behavioral innovation, which is regarded as a more direct variable reflecting the ability of birds to adapt to new environments.[13] Furthermore, there has long been concern and debate regarding the use of brain size as a predictor of animal behavior because contradictory results have been observed in multiple comparative studies.[38] Indeed, we did not find a significant correlation between any of three foraging innovation variables and brain size for 144 alien bird species with available brain size data (Pearson correlation coefficient, innovation rate versus brain size, $r = -0.046$, p = 0.58, n = 144; food innovation versus brain size, $r = -0.085$, p = 0.31, n = 144; technical innovations versus brain size, $r = -0.005$, p = 0.95, n = 144). We directly extracted the behavioral innovation data from Ducatez et al.[13] In that study, the authors built a unique database of >3,800 published field observations of birds that covered most regions of the world and documented the incorporation of new foods into the diet or the use of novel feeding techniques. Descriptions of the data are presented in Overington et al.[39,40] and Ducatez et al.[13] In brief, the database was compiled by systematically searching for reports of new behaviors in birds across 204 ornithology journals published between 1960 and 2018. A consumer innovation was defined as a feeding behavior if it was described in the report with key words such as novel, opportunistic, first description, not noted before, or unusual.[41] Similarly, a technical innovation refers to having a novel searching and handling technique regardless of whether the food type was novel.[13,39] For species with at least one recorded innovation, either consumer or technical, the total number of innovations reported was extracted and referred to as the innovation rate (the sum of consumer innovations and technical innovations; see the definition of innovation rate in Ducatez et al.[13]). Therefore, we used three variables of innovation in the present study: (1) innovation rate, (2) consumer innovations, and (3) technical innovations. Following Ducatez et al.,[13] we considered 8,645 species with these three innovation variables, including 7,397 species with zero innovation records and 1,248 species with at least one recorded innovation, for further analysis.

Other important variables affecting the establishment success rate

To further investigate the relationship between establishment success and innovativeness, we also considered important ecological and life-history traits that have been found to affect the establishment success of alien birds according to Redding et al.[12] from three sets of variables: (1) invasion-event-level factors (e.g., propagule pressure), (2) species-level factors (e.g., life-history traits), and (3) location-level factors (e.g., environmental similarity with the native range). For this study, we selected variables from all three sets of factors listed in Redding et al.[12] for which the effect sizes for establishment success ranked at least 10th among all factors and that were representative of those three sets of factors. By doing so, five key variables with significant effect sizes for the establishment success of alien birds and representative of these three sets of factors (i.e., including at least one key factor from each set of factors) were included in the present study. These five variables are as follows: (1) established populations (represents specific invasion-event-level factors), which is the number of alien taxonomic groups that are already established at a location at the time of introduction. This variable has an impact on establishment success because the ecological disruptions that are caused by (or enable) earlier invasions facilitate further successful introductions.[11] (2) Reproductive investment (represents species-level factors), for which we used brood size here, following previous studies. This variable is expressed as $\log_{10}(1/[(\text{broods per year}) \times (\text{reproductive lifespan})])$ and represents investment in

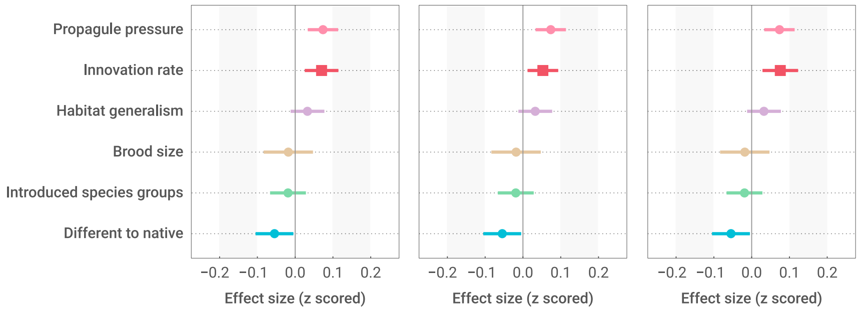

Figure 3. Parameter estimates (effect sizes) from the mixed-effects model of the success of alien bird establishment Shown are the estimate of each parameter and its 95% confidence interval. The effect sizes of three innovation variables are shown with box symbols, and those of other variables are shown with circle symbols.

the future over current reproduction.[4,12] (3) Propagule pressure (represents specific invasion-event-level factors), which is a numerical estimate of propagule pressure (founding population size), measuring both the number of introduction events per record (propagule number) and the number of individual birds that were introduced during each event (propagule size).[17,18] (4) Difference from native environment (represents location-level factors), which is the difference in environmental conditions between an invaded location and the native range of a species.[10] (5) Habitat generalism (represents species-level factors), which is a measure using the total number of different habitat use types.[42] Data on these five variables for 341 species were available and used for further analysis. For species trait factors, we did not include additional predictor variables, such as body size or body weight, that are not key traits for introduced avian invasion.[43]

Statistical analyses

We carried out all analyses within the R statistical environment.[44] We first combined all three datasets, which included data on the establishment success rate, behavioral innovation variables, and other important variables affecting the establishment success rate. This resulted in a total of 247 species (12,269 population records of invasion events, including 9,899 successful and 2,370 failed events) for which all data were available for further statistical analyses.

To quantify the association between the establishment success rate and behavioral innovation, we used linear mixed-effects models with multiple regression accounting for phylogenetic autocorrelation as implemented with the function phylolm in the package phylolm.[15] All models were run across a sample of 1,000 phylogenies using the Ericsson backbone obtained from www.bridtree.org[45] to account for phylogenetic uncertainty (coded as a random effect in the models), and the results represent an average across these. The linear mixed-effects model has been regarded as a robust multivariate analysis technique and is a widely used approach for comparative studies that can account for both phylogenetic autocorrelation and random effects to control the sampling pseudoreplication issue.[46] The establishment success rate was included as the response variable in the models. For the fixed effects, we fitted the innovation rate and the other five predictor variables listed above. To further investigate which type of innovation specifically affects the establishment success rate, we ran the model two additional times by replacing the innovation rate with consumer innovations and technical innovations, respectively. We calculated the standardized effect size for each of the predictor variables with a mean of zero and standard deviation to compare their relative importance in explaining the establishment success of alien birds.[12]

REFERENCES

1. Seebens, H., Blackburn, T.M., Dyer, E.E., et al. (2017). No saturation in the accumulation of alien species worldwide. Nat. Commun. ***8***, 14435.
2. Pysek, P., Hulme, P.E., Simberloff, D., et al. (2020). Scientists' warning on invasive alien species. Biol. Rev. ***95***, 1511–1534.
3. Blackburn, T.M., Pysek, P., Bacher, S., et al. (2011). A proposed unified framework for biological invasions. Trends Ecol. Evol. ***26***, 333–339.
4. Sol, D., Maspons, J., Vall-Llosera, M., et al. (2012). Unraveling the life history of successful invaders. Science ***337***, 580–583.
5. Sol, D., and Lefebvre, L. (2000). Behavioural flexibility predicts invasion success in birds introduced to New Zealand. Oikos ***90***, 599–605.
6. Sol, D., Duncan, R.P., Blackburn, T.M., et al. (2005). Big brains, enhanced cognition, and response of birds to novel environments. Proc. Natl. Acad. Sci. U S A ***102***, 5460–5465.
7. Sol, D. (2008). Do successful invaders exist? Pre-adaptations to novel environments in terrestrial vertebrates. In Biological Invasions, W. Nentwig, ed. (Springer), pp. 127–141.
8. Amiel, J.J., Tingley, R., and Shine, R. (2011). Smart moves: effects of relative brain size on establishment success of invasive amphibians and reptiles. PLoS One ***6***, e18277.
9. Dyer, E.E., Redding, D.W., and Blackburn, T.M. (2017). The Global Avian Invasions Atlas, a database of alien bird distributions worldwide. Sci. Data ***4***, 170041.
10. Pysek, P., Jarosik, V., Hulme, P.E., et al. (2010). Disentangling the role of environmental and human pressures on biological invasions across Europe. Proc. Natl. Acad. Sci. U S A ***107***, 12157–12162.
11. Simberloff, D. (2006). Invasional meltdown 6 years later: important phenomenon, unfortunate metaphor, or both? Ecol. Lett. ***9***, 912–919.
12. Redding, D.W., Pigot, A.L., Dyer, E.E., et al. (2019). Location-level processes drive the establishment of alien bird populations worldwide. Nature ***571***, 103–106.
13. Ducatez, S., Sol, D., Sayol, F., and Lefebvre, L. (2020). Behavioural plasticity is associated with reduced extinction risk in birds. Nat. Ecol. Evol. ***4***, 788–793.
14. Wiens, J.J., Ackerly, D.D., Allen, A.P., et al. (2010). Niche conservatism as an emerging principle in ecology and conservation biology. Ecol. Lett. ***13***, 1310–1324.
15. Ho, L.S.T., and Ane, C. (2014). A linear-time algorithm for Gaussian and non-Gaussian trait evolution models. Syst. Biol. ***63***, 397–408.
16. Reader, S.M., and Laland, K.N. (2002). Social intelligence, innovation, and enhanced brain size in primates. Proc. Natl. Acad. Sci. U S A ***99***, 4436–4441.
17. Lockwood, J.L., Cassey, P., and Blackburn, T.M. (2009). The more you introduce the more you get: the role of colonization pressure and propagule pressure in invasion ecology. Divers. Distrib. ***15***, 904–910.
18. Simberloff, D. (2009). The role of propagule pressure in biological invasions. Annu. Rev. Ecol. Evol. Syst. ***40***, 81–102.
19. Lockwood, J.L., Cassey, P., and Blackburn, T. (2005). The role of propagule pressure in explaining species invasions. Trends Ecol. Evol. ***20***, 223–228.
20. Bomford, M., Kraus, F., Barry, S.C., et al. (2009). Predicting establishment success for alien reptiles and amphibians: a role for climate matching. Biol. Invasions ***11***, 713–724.
21. Duncan, R.P., Bomford, M., Forsyth, D.M., et al. (2001). High predictability in introduction outcomes and the geographical range size of introduced Australian birds: a role for climate. J. Anim. Ecol. ***70***, 621–632.
22. Liu, C., Wolter, C., Xian, W., et al. (2020). Most invasive species largely conserve their climatic niche. Proc. Natl. Acad. Sci. U S A ***117***, 23643–23651.
23. Allen, W.L., Street, S.E., and Capellini, I. (2017). Fast life history traits promote invasion success in amphibians and reptiles. Ecol. Lett. ***20***, 222–230.
24. Jackson, M.C. (2015). Interactions among multiple invasive animals. Ecology ***96***, 2035–2041.
25. Liu, X., Wang, S., Ke, Z., et al. (2018). More invaders do not result in heavier impacts: the effects of non-native bullfrogs on native anurans are mitigated by high densities of non-native crayfish. J. Anim. Ecol. ***87***, 850–862.
26. Ballari, S.A., Kuebbing, S.E., and Nuñez, M.A. (2016). Potential problems of removing one invasive species at a time: a meta-analysis of the interactions between invasive vertebrates and unexpected effects of removal programs. PeerJ ***4***, e2029.
27. Adams, M.J., Pearl, C.A., and Bury, R.B. (2003). Indirect facilitation of an anuran invasion by non-native fishes. Ecol. Lett. ***6***, 343–351.
28. Darwin, C. (1859). On the Origin of Species (John Murray).
29. Sol, D., Lapiedra, O., and González-Lagos, C. (2013). Behavioural adjustments for a life in the city. Anim. Behav. ***85***, 1101–1112.
30. Médoc, V., Thuillier, L., and Spataro, T. (2018). Opportunistic omnivory impairs our ability to predict invasive species impacts from functional response comparisons. Biol. Invasions ***20***, 1307–1319.
31. D'antonio, C., Dudley, T., and Mack, M. (1999). Disturbance and biological invasions: direct effects and feedbacks. Ecosyst. World, 413–452.
32. Wang, X.Y., Yi, T., Li, W.H., et al. (2021). Anthropogenic habitat loss accelerates the range expansion of a global invader. Divers. Distrib. https://doi.org/10.1111/ddi.13359.
33. Diez, J.M., D'Antonio, C.M., Dukes, J.S., et al. (2012). Will extreme climatic events facilitate biological invasions? Front. Ecol. Environ. ***10***, 249–257.
34. Falconer, D.S., and Mackay, T.F.C. (1996). Introduction to Quantitative Genetics (Longman).

35. Davidson, A.M., Jennions, M., and Nicotra, A.B. (2011). Do invasive species show higher phenotypic plasticity than native species and, if so, is it adaptive? A meta-analysis. Ecol. Lett. ***14***, 419–431.
36. Lapiedra, O., Schoener, T.W., Leal, M., et al. (2018). Predator-driven natural selection on risk-taking behavior in anole lizards. Science ***360***, 1017–1020.
37. Wang, H., Wang, P., Zhao, X., et al. (2021). What triggered the Asian elephant's northward migration across southwestern Yunnan? The Innovation ***2***, 100142.
38. Healy, S.D., and Rowe, C. (2007). A critique of comparative studies of brain size. P. Roy. Soc. B Biol. Sci. ***274***, 453–464.
39. Overington, S.E., Morand-Ferron, J., Boogert, N.J., et al. (2009). Technical innovations drive the relationship between innovativeness and residual brain size in birds. Anim. Behav. ***78***, 1001–1010.
40. Overington, S.E., Griffin, A.S., Sol, D., et al. (2011). Are innovative species ecological generalists? A test in North American birds. Behav. Ecol. ***22***, 1286–1293.
41. Lefebvre, L., Whittle, P., Lascaris, E., et al. (1997). Feeding innovations and forebrain size in birds. Anim. Behav. ***53***, 549–560.
42. Simpson, E.H. (1949). Measurement of diversity. Nature ***163***, 688.
43. Jeschke, J.M., and Strayer, D.L. (2006). Determinants of vertebrate invasion success in Europe and North America. Glob. Change Biol ***12***, 1608–1619.
44. R Core, T. (2020). R: A Language and Environment for Statistical Computing (R Foundation for Statistical Computing). www.R-project.org.
45. Jetz, W., Thomas, G.H., Joy, J.B., et al. (2012). The global diversity of birds in space and time. Nature ***491***, 444–448.
46. Bolker, B.M., Brooks, M.E., Clark, C.J., et al. (2009). Generalized linear mixed models: a practical guide for ecology and evolution. Trends Ecol. Evol. ***24***, 127–135.

ACKNOWLEDGMENTS

We thank three anonymous reviewers for constructive comments that greatly improved this manuscript. X.L. was supported by grants from the Youth Innovation Promotion Association of the Chinese Academy of Sciences (Y201920) and the National Science Foundation of China (31870507). D.W. was supported by the CAS Pioneer Hundred Talents program.

AUTHOR CONTRIBUTIONS

X.L. and D.W. designed the study. X.L. and D.W. collected the data. D.W. and X.L. analyzed the data. X.L. and D.W. wrote the article.

DECLARATION OF INTERESTS

The authors declare no competing interests.

LEAD CONTACT WEBSITE

http://sourcedb.ioz.cas.cn/zw/zjrc/201603/t20160309_4546750.html.

SUPPLEMENTAL INFORMATION

Supplemental information can be found online at https://doi.org/10.1016/j.xinn.2021.100167.

The Innovation, Volume *2*

Supplemental Information

Behavioral innovation promotes alien bird invasions

Daiping Wang and Xuan Liu

Supplemental Information

Table S1. Linear mixed-effects model testing associations between establishment success rate and innovation along with other variables based on 247 species. Here, note that 'innovation rate' (i.e., the sum of 'consumer innovations' and 'technical innovations') is added as a fixed effect.

	Estimate		
	(β ± SE)	t	p
Random effects:			
Phylogeny (n =247)	0.61 (0.34-0.76)		
Fixed effects:			
Intercept	0.566 ± 0.146	3.9	——
Innovation rate	0.070 ± 0.023	3.1	0.002
Established populations	-0.019 ± 0.024	-0.8	0.435
Reproductive investment	-0.018 ± 0.033	-0.5	0.587
Propagule pressure	0.074 ± 0.021	3.6	<0.001
Different to native	-0.055 ± 0.026	-2.1	0.033
Habitat generalism	0.033 ± 0.023	1.4	0.154

Table S2. Linear mixed-effects model testing associations between establishment success rate and innovation along with other variables based on 247 species. Here, note that ‘Consumer innovations’ (i.e., the number of novel feeding behaviors) is added as a fixed effect.

	Estimate		
	(β ± SE)	t	p
Random effects:			
Phylogeny (n =247)	0.61 (0.34-0.76)		
Fixed effects:			
Intercept	0.567 ± 0.147	3.9	——
Consumer innovations	0.053 ± 0.021	2.5	0.012
Established populations	-0.022 ± 0.024	-0.9	0.369
Reproductive investment	-0.018 ± 0.034	-0.5	0.601
Propagule pressure	0.073 ± 0.021	3.5	<0.001
Different to native	-0.058 ± 0.026	-2.3	0.025
Habitat generalism	0.030 ± 0.023	1.3	0.197

Table S3. Linear mixed-effects model testing associations between establishment success rate and innovation along with other variables based on 247 species. Here, note that ‘Technical innovations’ (i.e., the number of novel searching and handling technique behaviors) is added as a fixed effect.

	Estimate (β ± SE)	t	p
Random effects:			
Phylogeny (n =247)	0.61 (0.33-0.76)		
Fixed effects:			
Intercept	0.565 ± 0.145	3.9	——
Technical innovations	0.076 ± 0.024	3.2	0.0018
Established populations	-0.016 ± 0.024	-0.7	0.515
Reproductive investment	-0.021 ± 0.033	-0.6	0.526
Propagule pressure	0.077 ± 0.020	3.7	<0.001
Different to native	-0.053 ± 0.026	-2.1	0.04
Habitat generalism	0.037 ± 0.023	1.6	0.102
